# Effects of Bovine Immunoglobulins on Immune Function, Allergy, and Infection

**DOI:** 10.3389/fnut.2018.00052

**Published:** 2018-06-22

**Authors:** Laurien H. Ulfman, Jeanette H. W. Leusen, Huub F. J. Savelkoul, John O. Warner, R. J. Joost van Neerven

**Affiliations:** ^1^FrieslandCampina, Amersfoort, Netherlands; ^2^Laboratory for Translational Immunology, University Medical Center Utrecht, Utrecht, Netherlands; ^3^Wageningen University & Research, Cell Biology and Immunology, Wageningen, Netherlands; ^4^Allergy Consortium Wageningen, Wageningen, Netherlands; ^5^National Institute of Health Research, Collaboration for Leadership in Applied Health Research and Care for NW London, Imperial College, London, United Kingdom

**Keywords:** bovine immunoglobulins, milk, colostrum, immune, infection, allergy

## Abstract

This review aims to provide an in depth overview of the current knowledge of the effects of bovine immunoglobulins on the human immune system. The stability and functional effects of orally ingested bovine immunoglobulins in milk products are described and potential mechanisms of action are discussed. Orally ingested bovine IgG (bovine IgG) can be recovered from feces, ranging from very low levels up to 50% of the ingested IgG that has passed through the gastrointestinal tract. In infants the recovered levels are higher than in adults most likely due to differences in stomach and intestinal conditions such as pH. This indicates that bovine IgG can be functionally active throughout the gastrointestinal tract. Indeed, a large number of studies in infants and adults have shown that bovine IgG (or colostrum as a rich source thereof) can prevent gastrointestinal tract infections, upper respiratory tract infections, and LPS-induced inflammation. These studies vary considerably in target group, design, source of bovine IgG, dosage, and endpoints measured making it hard to draw general conclusions on effectiveness of bovine immunoglobulin rich preparations. Typical sources of bovine IgG used in human studies are serum-derived IgG, colostrum, colostrum-derived IgG, or milk-derived immunoglobulins. In addition, many studies have used IgG from vaccinated cows, but studies using IgG from nonimmunized animals have also been reported to be effective. Mechanistically, bovine IgG binds to many human pathogens and allergens, can neutralize experimental infection of human cells, and limits gastrointestinal inflammation. Furthermore, bovine IgG binds to human Fc receptors which, enhances phagocytosis, killing of bacteria and antigen presentation and bovine IgG supports gastrointestinal barrier function in *in vitro* models. These mechanisms are becoming more and more established and explain why bovine IgG can have immunological effects *in vivo*. The inclusion of oral bovine immunoglobulins in specialized dairy products and infant nutrition may therefore be a promising approach to support immune function in vulnerable groups such as infants, children, elderly and immunocompromised patients.

## Human immunoglobulins in milk

The first year of life is essential for infants to develop their immune system. At the same time the child is exposed to many new and potentially immunogenic stimuli such as dietary components (food) allergens and the microbiota that colonize the gastrointestinal tract. Therefore, during this year, it is crucial to have tightly regulated immune responses to prevent inflammatory responses to these (stimuli) food antigens, allergens, and commensal bacteria.

It is known that human breast milk and bovine milk contain many factors that are involved in immune development. Milk contains immunoglobulins, prebiotic oligosaccharides, extracellular vesicles (e.g., exosomes), antimicrobial proteins such as lysozyme, lactoperoxidase, and lactoferrin, also immunomodulatory cytokines such as IL-10 and TGF-β. In addition, breastfeeding has been shown to provide protection against gastrointestinal and respiratory tract infections (1, 2). It prevents excessive immune responses during immune development in early life (3) which may be attributed to the orchestrated activity of the above mentioned milk components. Most of these components—with the exception of human milk oligosaccharides—are relatively well-conserved between bovine and human milk. Bovine milk and its components have clear functional effects on the human immune system (4). However, as many other immunomodulatory molecules are present in milk, the individual contribution of each of these to disease prevention is difficult to determine (5).

As early as 1892, Paul Ehrlich demonstrated that mouse maternal immunoglobulins confer passive immunity to their murine offspring (6, 7). Immunoglobulins (or antibodies) are glycoproteins produced by plasma/B cells that specifically recognize and bind antigens, that are present on e.g. bacteria and viruses. As the immunoglobulins have a high degree of specificity they assist in the destruction of specific pathogens. In addition to this specific recognition, various human immunoglobulin isotypes (IgM, IgG, IgA), and subclasses (IgG1-4, IgA1-2) differ in their biological features, structure, target specificity and distribution. Whilst IgG is the most predominant antibody in the serum, IgA is the main isotype of the immunoglobulins found in human milk and colostrum, followed by IgG and IgM.

In mature human breast milk, IgA is present at levels around 1 mg/ml, whilst in colostrum levels can reach up to 5–12 mg/ml (8, 9). The specificity of maternal IgA reflects the past exposure of the mother to bacteria and viruses present on mucosal surfaces in the gastrointestinal and respiratory tract and thus there is a transfer of immunological memory of the mother to the infant. Breastmilk IgA and IgG can promote neutralization and killing of pathogens and can also modulate immune function in infants, thereby protecting them against inflammatory responses (10–14). Therefore, the concept of vaccinating mothers against pathogens to optimize the protection of their nursing children has increasing interest (15, 16). Indeed in the UK both influenza and pertussis immunization are recommended during pregnancy and others such as for Group B streptococcal infection are now being tested *in vivo* (17).

The levels of serum IgA produced by the newborn infant itself are very low (18). At the age of 1 year, secretory (s)IgA is only 20% of the level observed in adults and circulating levels only reach maturity by the age of 4 years (18). Aberrant IgA responses in the gut have been described in several gastrointestinal diseases (19). Furthermore, delayed increases in IgA in the infant and low maternal IgA levels in breast milk have been associated with a higher risk of infection, allergy, and autoimmune diseases (20).

sIgA also has an important role in the development of oral tolerance toward the gut microbiota. Kohler showed that fecal sIgA concentrations peak at 1 month of age in exclusively breastfed infants compared to exclusively formula fed infants and decline to levels observed in formula fed infants around 5 months. From this timepoint onwards, no difference was observed between fecal sIgA concentration between breastfed and formula fed infants (21). In line with this, other studies showed that fecal sIgA concentration in exclusively formula-fed infants were only 33% of breastfed infants' levels at 1 month and only reached comparable levels to breastfed infants at 9 months of age [(21), and reviewed in (22)]. Low levels of fecal IgA in infants have been associated with a higher risk of allergy (23). Thus, the supply of sIgA in breast milk has a potentially pivotal role in enhancing intestinal immunity in early life and help in establishing a healthy outcome (24). However, interpreting the results of human observational studies of birth cohorts in relation to health outcomes is difficult as considerable variability exists in the breast milk content of immune active molecules both between mother/infant pairs and within such pairs at the different sampling points. Numerous genetic and environmental influences affect the levels of immune factors in milk and as yet no study has been able to account for the variability (22). Furthermore, as there are many other immunomodulatory molecules in the milk, the individual contribution of each to disease prevention is unknown (5).

Just as the multiple advantages of breast milk described, cow's milk consumption has also been associated with specific health outcomes. Several epidemiological studies have shown a positive association between the consumption of unprocessed cow's milk and a reduced risk for developing allergy, most prominently allergic asthma (25–27). This association was not found for the consumption of heated farm milk and commercial high heat treated milk (e.g., UHT) (28). The consumption of these different processed cow milk types is consumed on top of regular breastmilk and formula milk consumption and can thus be considered as weaning food. The levels of intact milk protein in these cow's milks correlated with the protective effect on asthma (28), suggesting that intact milk protein confer this effect but not denatured protein which occurs upon heating (29, 30). Several factors that are present in breast milk as well as in cow's milk can be linked to this effect on allergy (4). In addition, raw milk consumption in the first year of life is also associated with decreased risk of upper respiratory tract infections and otitis media (31).

This well-established protective function of immunoglobulins in breast milk, and in neonatal health in farm animals, has meant that bovine immunoglobulins have been studied extensively for their putative effect on human health.

## Bovine immunoglobulins from milk, colostrum, and serum

Bovine milk contains many components that have immunomodulatory and antimicrobial properties. Bovine immunoglobulins (bIg), in particular bovine IgG, have been studied since the 1970's for their potential effects on immunity and infection in humans.

Bovine IgG can not only bind to a wide range of pathogenic bacteria and viruses (32–40), but also to many allergens (41). In addition, the specificity of the immunoglobulins in the milk or colostrum can be increased by vaccinating the cows before collecting their milk or colostrum. After vaccination hyperimmune colostrum is strongly enriched for IgG1 that recognizes the pathogen included in the vaccine. Indeed, the first studies on the use of oral bovine immunoglobulins to prevent and treat gastrointestinal infections have focused on the use of hyperimmune colostrum containing rotavirus-specific immunoglobulins (40, 42). These were used for the treatment and prophylaxis of rotavirus infection in infants and children and showed good efficacy in several studies (described in section Treatment of Git Infection in Infants and Children and Prevention of Gastrointestinal Tract infection). Since these early studies, many studies have been performed utilizing different *in vitro* systems, animal models and human studies in order to determine mechanisms of action and their applicability for human health.

### Sources and origin of bovine IgG

In contrast to humans, in cow's milk IgG is the main isotype present, especially in colostrum, followed by IgA and IgM (43). There are 2 subclasses of IgG known in cows: IgG1 and IgG2. IgG1 is the main isotype of immunoglobulin found in bovine milk and colostrum. In mature milk IgG1 is the dominant isotype, whereas sIgA and IgM are present at ~5–10 fold lower levels. The concentration of IgG1 in mature milk is around 200–500 μg/ml although levels reported in literature differ depending on type of measurement technique used (44, 45).

In colostrum the IgG levels are much higher, reaching up to 50–100 mg/ml in the first days after birth [reviewed in (46)]. These high concentrations are necessary for the calf because cows cannot transfer IgG across the placenta. Calves are thus dependent on the transfer of IgG from colostrum into the blood, and calves that do not receive colostrum after birth are immunocompromised and prone to infection. High levels of colostrum-derived IgG in blood of calves are associated with reduced risks of pneumonia (47). Likewise, colostrum intake by calves is crucial to protect against gastrointestinal tract infections. This uptake of IgG occurs partly via passive transport across the epithelium when it is not yet fully closed, but also actively via FcRn, the neonatal IgG receptor that is expressed on intestinal epithelium in neonatal calves (48, 49).

This high level of IgG in colostrum has meant that many studies have used colostrum as an IgG rich source for studies, rather than purified IgG (1). However, in colostrum—as in mature milk—many other factors are present that can have immunological effects, but compared to IgG their concentrations in colostrum are relatively low. For this reason, colostrum is often regarded as an IgG1 preparation even though additional factors are present. More recently, bovine immunoglobulins isolated from serum—that is also high in IgG and low in IgM and IgA—have been used to study their effects on chronic and severe gastrointestinal disturbances in humans (50).

Maternal serum IgG levels in cows decrease before delivery (51), shown by following labeled IgG1 and IgG2 transfer from blood into colostrum (52). The majority of all maternal IgG in colostrum thus seems to be transported from the serum into colostrum and milk (52, 53). The immunoglobulins in milk and colostrum can therefore be considered as a good reflection of the immunoglobulins in maternal serum.

### Digestion of bovine IgG after oral administration

In order to be used as an active, prophylactic ingredient in human food products, a significant amount of bovine immunoglobulins must be able to survive passage through the stomach into the small intestine, or even throughout the entire gastrointestinal (GI) tract. This has been the subject of several studies, reviewed in detail in (54).

An early study by Hilpert et al. showed that Enteropathogenic *E. coli* (EPEC)-specific IgG or active fragments thereof are present in the stools of infants that received immunoglobulins from cows immunized against EPEC (55). In line with this, in the stools from children with rotavirus that received oral colostral immunoglobulins anti-rotaviral immunoglobulin activity could be detected (56). This data indicate that IgG can survive passage through the GI tract and retain functionality.

In a study of ileostomy patients, a total of 50% of all orally administered IgG1 from an bovine immunoglobulin concentrate could be recovered in ileal fluid (57). Interestingly, no enhanced survival was seen by combining the preparation with antacid or omeprazole (a proton pump inhibitor) to counter the effect of low gastric pH, suggesting that pH regulation in the stomach does not enhance the survival of the IgGs.

Two other studies have shown that up to 10–20% of the immunoglobulins may survive GI passage in infants (58) and adults (59). However another study, showed only 3% of orally ingested bovine IgG could survive the passage through the GI tract in healthy adults (60). Encapsulation of the immunoglobulins in entero-protective capsules that dissolve at pH 6 and higher, increased GI tract survival of bovine IgG from 3 to 30% (60). Similarly, in mice consuming bovine colostrum, 5% of the IgG could be recovered in the colon, which was increased to >20% for encapsulated colostrum (61).

In contrast, two other studies could not recover significant amounts of bovine IgG in stools of healthy adults (36, 62). Bogstedt (62) showed <0.01% of orally ingested bovine IgG recovery in stool in healthy adults. Furthermore, Lissner (36) showed low recovery and only in 3 out of 8 healthy volunteers after ingestion of 15 g of colostrum powder. It was noted that the longer residence time in the large intestine is determining the observed low survival of IgG, especially when compared to other studies in which people with diarrhea were investigated (62). Furthermore, a recent study showed that even though orally ingested bovine IgG can be detected in fecal matter, bovine IgG is not taken up into circulation in adult human volunteers (63). An explanation of the different recoveries of bovine IgG in fecal matter after oral administration by adults could rely on differences in time of fecal matter sampling, the analytical methods used or set up of the study. Kelly et al. (60) who showed the 3% recovery level tracked fecal passage by oral ingestion of carmin red combined witha 3 days stool collection. As the duration of fecel transfer differs per person, and if no tracker is used (36, 62), it is difficult to predict the peak of bovine IgG in feces. The risk is that the peak may be missed. Furthermore, differences in protease inhibitors have been reported therefore a cocktail (60) might be more effective compared to a single trypsin inhibitor (64).

Even though these findings consistently show survival of bovine IgG throughout the GI tract, the amounts recovered in feces vary from trace amounts (0.01%) up to 50% between different studies most likely due to differences in study methodology.

Collectively, the data indicates that a significant amount of orally ingested bovine IgG immunoglobulins are present throughout the GI tract, especially so in infants as they have a higher gastric pH and lower levels of proteolysis in the GI tract. The recent insights in the role of maternal breast milk immunoglobulins and its role in microbiota development in the infant stress the importance of keeping these structures intact. This is further discussed in section Immunological effects of Oral immunoglobulins of other species.

### Specificity of bovine IgG in normal milk and colostrum

The presence of bovine IgG1 against rotavirus in normal cow's milk was first demonstrated in the 1970's by Ellens et al. (37). This was followed by a series of studies on binding of bovine IgG to many human bacterial pathogens including *Yersinia enterocolitica, Campylobacter jejuni, Escherichia coli, Klebsiella pneumoniae, Serratia marescens, Salmonella typhimurium, Staphylococcus, Streptococcus, Cryptosporidium, Helicobacter, E.coli EHEC O157:H7, Pseudomonas*, and Rotavirus (32–36). Bovine colostrum also contains IgG1 and IgA antibodies directed against Necrotizing enterocolitis (NEC)-associated pathogens such as *Klebsiella, Citrobacter, Enterobacter* and *Serratia* (65).

In addition to recognizing gastrointestinal tract associated pathogens, bovine Igs can also bind to respiratory pathogens such as human Respiratory Syncitial Virus (RSV), influenza virus and *Streptococcus pneumoniae* (38). This may in part explain the finding by Loss et al. that raw milk consumption in the first year of life is also associated with fewer upper respiratory tract infections and otitis media (31).

In addition to immunoglobulins specific for infectious microorganisms, Collins et al. have shown that cow's milk and colostrum can also contain immunoglobulins that are specific for aeroallergens like rye-grass pollen, house dust mite, aspergillus, and wheat allergens (41).

Although bovine milk IgG has been investigated to a larger extent than IgA, it is assumed that the specificities are similar between the two. Furthermore, a recent report compared the specificity between bovine sIgA and human sIgA isolated from milk to pathogenic and commensal bacteria and concluded that the binding characteristics of both human and bovine sIgA from milk were similar (66).

**Figure 1:**
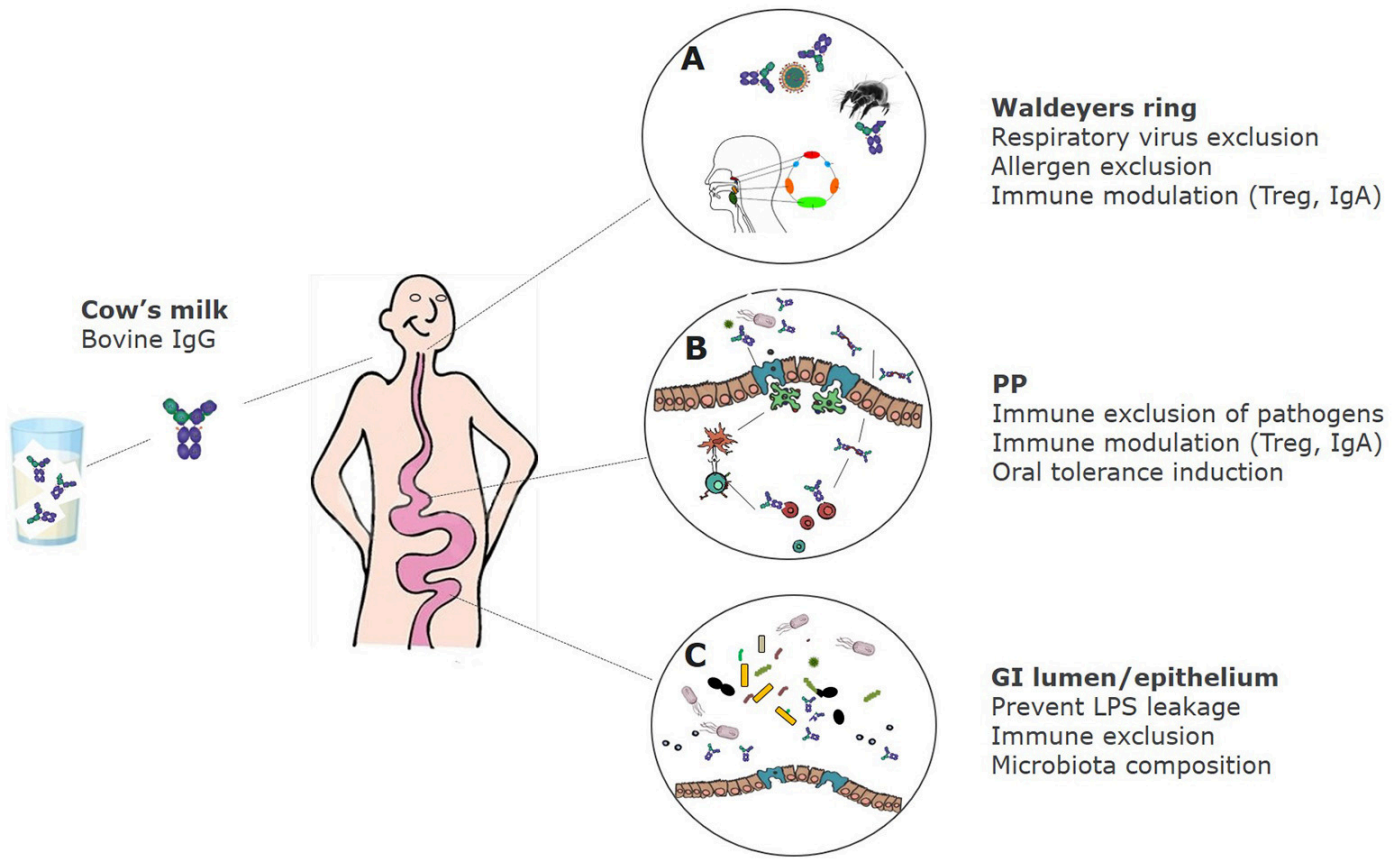
Proposed effects of bovine IgG at various locations in the GI tract. **(A)** After ingestion bovine IgG can encounter swallowed respiratory pathogens and inhaled allergens. This can lead to partial immune exclusion (especially when milk is regurgitated and enters the nasopharynx) and immune modulation in the tonsils that comprise Waldeyer's ring. **(B)** In the small intestine IgG can also exclude pathogens by preventing adhesion to epithelial surfaces, but may also promote the uptake of immune complexes of IgG with pathogens via Fc receptors, resulting in regulatory immune responses and induction of IgA. **(C)** In the colon, IgG prevents leakage of LPS, can modify microbiota composition and short-chain fatty acids (SCFA) production, and can prevent adhesion of pathogens.

In summary, bovine immunoglobulins can bind to a wide range of human intestinal and respiratory bacterial—as well as viral pathogens, and can also bind to inhalation and some food allergens. Figure 1 shows the proposed effects of bovine IgG along the gastrointestinal tract.

## *In vivo* studies on effects of bovine IgG and colostrum on gastrointestinal tract infections

Studies on the effect of bovine immunoglobulins on human immune function and susceptibility to infection have been performed with a wide range of Immunoglobulin rich products which can be categorized in three groups: IgG-isolates from colostrum or milk, IgG-rich colostrum, and serum-derived IgG. These products have either been tested for prophylactic or therapeutic effects in field settings or in controlled challenge models.

### Treatment of git infection in infants and children

A number of studies have been performed in which (hyperimmune) colostrum or immunoglobulins derived thereof were used for treatment in infants and young children and are summarized in Table 1. Four independent studies describe rotavirus-diarrhea treatment with hyperimmune colostrum products. Two studies were double blind, placebo controlled studies (67, 68) performed in Bangladesh and another two were controlled studies in Europe (58, 69). Three of the studies showed significant clinical effects including a reduction in duration of diarrhea (67) and stool frequency (67, 68) the duration of rotavirus excretion (58) and need for oral rehydration solution (ORS) (68). In the fourth study, a trend was noted toward shorter duration and decreased stool frequencies in the actively treated group, but were not significant compared to the control group (69). All these studies on treatment were performed in infants of a similar age (~4–30 months old) but with bovine immunoglobulin doses ranging from 3 g (100 ml colostrum) to 20 g (based on a 10 kg child receiving 2 g/kg body weight).

**Table 1:** Treatment of GIT infections in infants and children.

**Subject characterisitics**	**Product, dosage, and duration**	**Type of study**	**Reported outcome**	**References**
3 months−6 year Japanese children with rotavirus diarrhea (active *n* = 18/placebo *n* = 26)	Hyper immune colostrum (titer 1:1280 – 1:5120) 20–50 mL/day, 3 days	Randomized	No significant differences between Rota colostrum recipients and controls.	(40)
6–24 months old Bangladeshian children with rotavirus diarrhea (active *n* = 35/placebo *n* = 33)	Hyperimmune- vs. normal colostrum rota strains WA, RV5, RV3 and ST3. 100-fold titer difference 100 mL/day, 3 days	Randomized, double blind	Reduction of duration and severity of diarrhea in treatment group	(67)
4–24 months old Bangladeshian children with rotavirus diarrhea (active *n* = 40 placebo *n* = 40)	Hyper immune colostrum 4 × 2.5 g/day, 4 days	Double blind	Decreased stool output and -frequency; increased, and faster clearance of rotavirus from the stool	(68)
<2 year old German children with rotavirus diarrhea [acive *n* = 46; placebo *n* = 45 (low titer)] and [active *n* = 43; placebo *n* = 30 (high titer)]	Bovine antibody concentrate. Low and high rotatiters (low: f1.100 high: = 1:6.000) 2 g/kg bw/day, 5 days	Open controlled	Low titers, no effect. High titers; trend for reduced duration diarrhea, sign. reduction viral shedding.	(58)
6–30 months old Finnish children with rotavirus diarrhea (*n* = 42; *n* = 42; *n* = 41)	Hyperimmune- vs. standard colostrum vs. milk SA rotavirus titers: 1:597 vs. 1:128 vs. 1:16 4 ×100 mL/day, 4 days	Randomized, double blind	Trend but no significant differences for weight gain, reduced duration and frequency of diarrhea in hyperimmune group compared to other 2 groups	(69)
10 day−18 months German children with ETEC diarrhea (*n* = 60)	Hyperimmune milk (0–7 days) 0.32 g/kg/day 1 g/kg, 10 days	Open	Negative stool culture in 84% of the patients	(70)
4–24 months Bangladeshian children with a history of acute watery diarrhea for ‘48 h (active *n* = 32; placebo *n* = 31)	Hyperimmune- vs. non-immune milk Ig against EPEC and ETEC, 45, 40% respectively. 20 g/day, 4 days	Placebo controlled, randomized, double blind	No effect of hyperimmune milk on any of the outcomes.	(71)
4–29 months old *H. pylori* infected Bangladeshian children (active *n* = 12;placebo *n* = 12)	Hyperimmune Ig preparation vs. non-immune colostrum, 1 g/day, 30 days	Placebo controlled, double blind pilot	No effect was observed by hyperimmune product on H pylori infection as determined by UBT	(72)
1–12 year old Bangladeshian children with bloody mucoid diarrhea <5 days, Shigellosis (active *n* = 34; placebo *n* = 35)	Hyperimmune vs. non-immune colostrumtiter 3,200–6,400 anti-shigella 3 × 100 mL, 3 days	Placebo controlled, randomized	No difference between hyper and non-immune colostrum group in any of the outcomes	(73)
1 months−18 year old German children with *E. coli* associated diarrhea (active *n* = 13;placebo *n* = 14)	Immunoglobulin concentrate (Lactobin) vs. placebo 80% protein, >65% Immunoglobulin 3 × 7 g, 14 days	Placebo controlled, double blind	Stool frequency was reduced in Ig Concentrate treated group. No effects on carriage pathogens and complications of infections.	(74)

An early treatment study providing a specific milk immunoglobulin concentrate (1 g/kg/day for 10 days) against enteropathogenic *E. coli* (EPEC) to infants (10 days−18 months old) suffering from enteropathogenic *E. coli* (EPEC) induced diarrhea showed stool cultures being negative for EPEC in 84% of the cases (70). The control group suffered from diarrhea induced by other *E. coli* strains that did not receive the milk immunoglobulin concentrate and resulted in negative stool cultures in only 11% (1/9) of the cases. In another, double blind, placebo controlled study, performed in Bangladesh Casswall et al. could not demonstrate a significant effect of an oral bovine immunoglobulin milk concentrate from cows hyperimmunized with enterotoxicogenic *E. coli* (ETEC) and EPEC strains on the duration, ORS intake, stool frequency or duration of *E.coli*-induced acute diarrhea (71). Within this study, children (4–24 months of age) that already had *E. coli* induced diarrhea were included and given 20 g of immunoglobulin concentrate for 4 days. This is a 20-fold higher dose than described in the study by Mietens et al. (70). Since no effects were seen in the Bangladesh study, it was hypothesized that the strains used for vaccination of the cows and the strains that infected the children differed too much.

For other pathogens such as *Helicobacter pylori* and *Shigella*, the use of hyperimmune colostrum in treatment has been less successful compared to rotavirus and *E. coli* induced diarrhea. In a study performed in infants in rural Bangladesh *Helicobacter pylori*-infected children (4–29 months of age) treatment with purified immunoglobulins from hyperimmune colostrum failed to eradicate *H. pylori* infection (72). In this double blind, placebo controlled study, children were treated for 1 month with 1 g hyperimmune colostrum containing 0.7 g of immunoglobulins per day. Although hyperimmune colostrum against *Helicobacter pylori* prevented adhesion of helicobacter *in vitro*, and treated an infection *in vivo* in mice (75), an effect *in vivo* in humans was not observed. Specificity of the strains used for the vaccine or age of the target group are two factors that might be responsible for the differing outcomes. Hyperimmune colostrum of cows immunized with *Shighella* did not have an effect on *Shighella* dysenteria-infected children (1–12 years of age) in Bangladesh in a double blind, placebo controlled trial (73). No significant differences were seen in duration of diarrhea, fever, stool frequency and visible blood in the stool between the actively treated (100 ml of hyperimmune colostrum 3 times per day) and the control group. Interestingly, a single placebo controlled study reported that children (0–18 years, mean age of 2) suffering from Shiga toxin-producing *E. coli* induced diarrhea and treated with a normal (non-hyperimmune) colostrum resulted in significantly reduced stool frequency (74). Yet, no effects were shown on pathogen numbers or complications of infection. One of the factors which might explain the difference in the observed effect was the timing of the start of treatment. In the study that did show a beneficial effect of treatment was started 24–48 h after diarrhea and in the other study only after 48–72 h. Other reasons such as study power cannot be excluded. Intervention studies have been described in literature testing immunoglobulin preparations for treatment and/or prevention of parasites and fungi reviewed by Hammarstrom et al. (76).

Although not all studies discussed above show effects of bovine IgG in the treatment of on-going gastrointestinal tract infections in children and infants, the majority of the studies indicate that bovine IgG, especially after prior bovine vaccination with the specific organism, can help in shortening the duration of gastrointestinal infections.

### Treatment of gastrointestinal infections in adults with hyperimmune colostrum

In a single, human trial with adult patients suffering from *Clostridium difficile* associated diarrhea, Van Dissel et al. (77) showed that a whey protein concentrate (WPC40) from *Clostridium* immunized cows containing a high concentration of specific sIgA might prevent relapsing clostridium-induced diarrhea after antibiotic treatment. The trial was uncontrolled, and should therefore be considered as preliminary. In addition, the study also showed that the hyperimmune WPC40 could prevent lethality in a hamster model of *Clostridium* infection.

Similarly, in a prospective randomized double blind study to compare treatment of *Clostridium difficile* associated diarrhea patients were treated with either colostral immunoglobulins or metronidazole. The study showed that colostral immunoglobulins were as effective in the prevention of *clostridium difficile*-associated diarrhea as treatment with metronidazole (78).

A recent trial treating *Helicobacter pylori*-infected adults with milk of immunized cows (randomized double-blind trial) showed an increased clearance of intragastric *Helicobacter pylori* (79). A third gastrointestinal pathogen that has been studied for its' potential to be treated with bovine hyperimmune colostrum is *cholera*-induced diarrhea. In this paper the results of two randomized, controlled clinical trials were reported but no effect on reducing stool volumes was observed (80).

### Prevention of gastrointestinal tract infection upon a pathogen challenge of healthy volunteers

To assess the biological activity of new nutritional concepts, studies have been set up that utilize adult human volunteers that were given immunoglobulin preparations derived from hyperimmune colostrum orally before a challenge with live pathogens. These studies are mainly performed with products containing immunoglobulins specific for *E.coli* to prevent travellers' diarrhea.

In the first study, Tacket et al. (81) showed a clear prophylactic effect of hyperimmune colostrum from cows immunized with a range of *E.coli* serotypes. In this study volunteers received an oral challenge with 10^9^ enterotoxicogenic *E.coli*. The volunteers received either hyperimmune colostrum (3 times/day) or a control immunoglobulin concentrate without *E. coli*-specific immunoglobulins. None of the volunteers in the hyperimmune colostrum, while 9/10 in the control group developed diarrhea. Similar preventive effects of *E.coli* specific immunoglobulins were reported by Freedman et al. (82). However, in a follow-up study no effect could be seen using an enteric coated *E.coli* specific immunoglobulin product (83). It could be that the immunoglobulins in enteric capsules were not released until they reached the colon, whilst the infection is initiated in the small intestine (60).

In a placebo controlled challenge study with ETEC H10407, oral hyperimmune bovine IgG isolated from hyperimmune colostrum protected against ETEC-induced diarrhea (84). In a challenge model with an 078 ETEC strain, colostrum protein formulated in tablet form was shown to confer protection against diarrhea in two separate randomized, double blind placebo controlled studies (85).

In a similar challenge study, healthy volunteers were infected with *Cryptosporidium parvum* and prophylactically treated with a hyperimmune colostrum (86). In this study only a non-significant trend toward less diarrhea was noted in the treated group.

The treatment of GI-tract infections in adults, the prophylactic approach in challenge studies with healthy volunteers indicates that hyperimmune immunoglobulins can alleviate or shorten GI tract infections in a well-controlled challenge setting.

### Prevention of gastrointestinal tract infection

It was first shown in the 1960s that bovine colostral immunoglobulins protected calves against gastrointestinal bacterial and viral infections (87, 88). This has prompted researchers to investigate the efficacy of bovine immunoglobulins in humans. Alarge number of studies have now shown efficacy of bovine immunoglobulins in preventing natural infection in humans as well as in animal infection models (76). Studies on prevention of gastrointestinal tract infections in infants are summarized in Table 2. This concept has been tested extensively for anti-rotavirus antibodies. One of the first studies to demonstrate functional effects of bovine immunoglobulins in humans was a study by Ebina et al. (40) showing that bovine colostrum from naturally rotavirus infected cows protected young children against rotavirus diarrhea during a rotavirus epidemic. A similar protective effect of rotavirus-specific bovine colostral immunoglobulins was described by Davidson in a controlled study in hospitalized Australian children aged 3–15 months (42). In a later study, Davidson confirmed this in Hong Kong and India (89). However, an infant formula with a bovine milk immunoglobulin concentrate from cows immunized with *E. coli* EPEC and Rotavirus did not protect against the development of diarrhea in a controlled study carried out in Chile (91). The reason of the lack of efficacy is not known but the authors speculated that the dose (0.12 g/kg/day) might have been too low. Specificity of the antibodies was in line with the observed pathogens in the study and therefore an unlikely reason for the observed lack of effectivity. Yet, a preventive effect of bovine colostrum immunoglobulins of cows immunized with rotavirus was shown in a study in 3–7 months of old infants in America (90). This was also shown for hyperimmune colostrum against E.coli in a study in 3–6 month old infants in Iraq (92). Infants in the active group had significantly lower incidence in diarrhea and episodes of diarrhea were shorter in duration during a 6 months follow up period. In this study, no effect was seen in children receiving colostrum immunoglobulins from non-immunized cows implying that specific antibodies were needed for the observed benefit. Indeed, rotavirus-specific IgG1 immunoglobulins from bovine milk were shown to protect mice from rotavirus-induced diarrhea (39). Similar findings were reported by others in rotavirus infection models in mice (95, 96), cows (97, 98), and piglets (99). The bovine immunoglobulins were also shown to functionally inhibit the replication of human rotaviruses in *in vitro* tissue culture experiments. This protective effect of hyperimmune bovine IgG has not only been demonstrated for rotavirus in *in vivo* animal models but also for other pathogens such as enteropathogenic *E. coli* bacteria (100) and *Helicobacter pylori* (72, 75).

**Table 2:** Prevention of Gastrointestinal tract infections in infants.

**Age of infants /children**	**Product, dosage, and duration**	**Type of study**	**Reported outcome**	**References**
3 months–6 year old Japanese infants (active *n* = 18; control *n* = 26) and 1 month–3 year old Japanese orphaned infants (active *n* = 6; control *n* = 7)	Hyperimmune colostrum 0.6 g IgG + 0.02–0.05 g IgA/day 20 mL	Randomized	Prevention of rota infection but no effect on duration of diarrhea, bowel movements or virus shedding in stool.	(40)
3–15 months old Australian infants (active *n* = 55; control *n* = 56)	Hyperimmune colostrum 50 mL/ 1 day, 10days	Randomized, controlled	Effective; 9/ 65 control children and 0/55 treated children acquired rotavirus infection	(42)
1–36 months old infants from HongKong and India (actve *n* = 50; placebo *n* = 102)	Hyperimmune colostrum 2 g/day. 3x a day hospital stay + 3days after	Randomized double blind	0/23, 0/27 in treated and 5/50 and 8/52 in control group showed rota infection in India and HK resp.	(89)
3–7 months old American infants (actve *n* = 31; control *n* = 33)	Hyperimmune colostrum (0.2 mg/ml in formula) >360 mL formula/day, max 6 months	Randomized, controlled	No. of days with diarrhea and rota associated diarrhea sign lower in treatment group. Incidence not sign lower.	(90)
3–6 months old Chilean children (active *n* = 124; control *n* = 108)	Milk Ig concentrate, immunized cows 1 g/day 0.5% wt/wt rota + 0.5% wt/wt EPEC immune conc. 6 months	Double blind	No effect on incidence nor duration of diarrhea	(91)
3–6 months old Iraqi infants (*n* = 125)	Hyperimmune colostrum 0.5 g/kg bw	Randomized double blind	Lower incidence of diarrhea	(92)
1–6 year old Egyption children with recurrent URTI and/or diarrhea (GITI) (*n* = 160)	Bovine colostrum 3 g/day for <2 year 6gr/day for >2 years, 4 weeks	Open, non-comparative	Lower number of episodes and hospitalizations for GITI (and URTI)	(93)
1–8 year old Indian children with recurrent URTI and/or diarrhea (*n* = 605)	Bovine colostrum 3gr/day, 12 weeks	Open, non-comparative	Lower number of episodes and hospitalizations for GITI (and URTI)	(94)

Preventive studies do not always focus on a specific pathogen to induce diarrhea. In fact, scoring disease episodes within a specific time is a commonly used approach to test whether a specific product lowers the incidence of the disease. Two studies from India and Egypt used this approach and have shown decreased diarrhea in children that received (non-hyperimmune) colostrum products (93, 94). Both studies included children with recurrent infections prior to the start of the study, but unfortunately as both were uncontrolled the colostrum products were not compared to a placebo. Therefore, these results should be interpreted with caution.

The strongest evidence on protective effects of bovine immunoglobulins from non-immunized cows comes from studies in HIV patients with recurrent diarrhea (32, 101–106) and are summarized in Table 3. As HIV patients are strongly immunosuppressed as a result of the depletion of CD4+ T cells, they have diminished capacity to resist infections and are highly susceptible to diarrhea, especially induced by *Cryptosporidium, Amoeba* and *Campylobacter*. The interest in colostrum for treating HIV infected people came from an initial report that described a positive effect of an immunoglobulin preparation derived from colostrum of non-immunized cows (32, 101). This was confirmed in another study using a colostrum-based porridge (103). Stool frequencies in these studies decreased from ~7 times/24 h period to 1–3 times/24 h. In addition to reduced stool frequency, decreased fatigue scores and increased weight and CD4+ T cell counts were also noted (104–106).

**Table 3:** prevention of GIT in HIV patients.

**Number of subjects**	**Country of study**	**Product, dosage, and duration**	**Type of study**	**Outcome**	**References**
*n* = 37	Germany	Igs from bovine colostrum (Lactobin) 10 g/day, 10 days	Non-controlled pilot study	Mean daily stool frequency decreased from 7.4 to 2.2 at the end of the treatment	(32)
*n* = 1	UK	Igs from bovine colostrum (Lactobin) 50 g/day, 14 days	Case report	Clinical improvement of diarrhea and elimination of parasite.	(101)
*n* = 25	Germany	Igs from bovine colostrum 10 g/day, 10 days, 20 g/day 10 days in non-responders	Prospective, open, uncontrolled	Complete (40%) or partial (24%) remission of diarrhea in 64% of the patients	(102)
*n* = 30	Nigeria	Bovine colostrum product (ColoPlus) 2 × 50 g/day, colostrum 32% containing 3–4 g IgG /50 g	Open label observational study	Reduced nr defecations/day. Increase haemaglobulin, albumin. Alleviated fatique, increased CD4+ cells.	(103)
*n* = 8	America	Serum derived bovine Ig 2.5 g/day, 8 weeks	Open label	Improvement in symptoms with reduced bowel movements/day (*P* = 0.008) and improvements in stool consistency (*P* = 0.008)	(104)
*n* = 850	Uganda	Colostrum based food	Field trial	Improved nutritional and immune status (increased body weight, decreased fatigue, transient rise in CD4 Tells)	(105)
Active *n* = 45; control *n* = 42	Uganda	Colostrum based supplement 2 × 50 g/day, 4 weeks	Randomized single-blind controlled trial	Daily stool frequency decraeased by 79% during study period in colostrum group compared to 58% in control group(*p* < 0.001)	(106)

These studies clearly demonstrate that the administration of passive immunity in the form of bovine immunoglobulins can be protective against a range of pathogens and is especially effective in immune compromised individuals. Interestingly, overall it seems that treatment and prevention of rotavirus infection with bovine IgG from vaccinated cows is more effective than the same approach for pathogenic bacteria. However, in bacterial challenge models, bovine IgG of vaccinated cows is more effective against bacterial pathogens than in prevention and treatment of normal infections. In these challenge models the pathogen with which the volunteers are challenged is the very same bacterial strain that is used for vaccination of the cows. As a result it is expected that the “coverage” of bacterial epitopes by the IgG raised in the cows is complete, and therefore effective in subsequent challenge models. In a field trial this coverage might not be always complete with the result that the bacteria still finds a way to resist the limited action of the antibodies. Indeed, bacteria have evolved to escape the immune system (107, 108).

In relation to treatment and prevention of natural infections by rotavirus, we speculate that the virus has fewer surface proteins and (oligo)saccharides involved in adhesion and infection than more complex bacterial pathogens. As the prevention and treatment is done with IgG raised against different strains of rotavirus and bacteria than the ones that may be actually the cause of infection, the chance of cross protection of bovine IgG to other strains of similar pathogens is better for rotavirus than for bacteria. This could be because of the more limited numbers of relevant cell surface antigens that are needed to initiate infection with rotavirus.

## *In vivo* studies of breastfeeding and bovine IgG and colostrum on respiratory tract infections and the relation with allergy

### Breastfeeding and prevention of respiratory tract infections: a link to asthma?

Respiratory tract infections can be divided into upper respiratory tract infections (URTI) and lower respiratory tract infections (LRTI). Especially URTI are very common in childhood. Likewise, otitis media, which is infection of the middle ear is highly prevalent, and has partial overlap in symptoms with URTI. Multiple cohort studies have shown that breastfed infants have a lower risk to develop respiratory infections and otitis media compared to formula fed infants (1–3, 109, 110). Duijts (111) et al. showed that exclusive breastfeeding up to 6 months tended to be more protective for upper and lower respiratory tract infections compared to 4 months exclusive breastfeeding. Yet for recurrent upper respiratory infections defined as >3 episodes of influenza/cold Chantry (2) et al. did not observe a difference between not breastfed, <1 months, 1–3 months, 4–5 months or >6 months breastfed infants. Interestingly, all groups that were breastfed for <6 months had a 2-fold higher chance to develop recurrent otitis media compared to infants that were breastfed for >6 months. In line with this Ip et al. showed in a meta-analysis of 5 studies that breastfeeding was associated with a reduced risk of otitis media (1). In summary, it is not clear what the optimal duration and amount of breastfeeding is for protection against these infections. The effects found differ between studies and between types of outcome (pneumonia, upper vs. lower respiratory infections, and otitis media). Much of the variation could be attributed to the impact of gene/environment interactions having diverse effects on levels of immune-modulatory molecules in breast milk between study subjects (112, 113).

There is a complex relationship between early respiratory tract infection and the subsequent evolution of allergy [Summarized in (114)]. The previously labeled hygiene hypothesis suggested that early infection was associated with less subsequent allergy and allergic disease, most notably asthma. There is a credible immunological mechanism to explain this mutually exclusive phenomenon in that interferon gamma (IFN-γ) generated during infection down-regulates T-helper lymphocyte-2 (Th2) activity associated with the production of interleukin-4 (IL-4) which promotes IgE production. In addition infection also up-regulates T-regulatory cell (T-reg) activity, which has additional controlling effects on Th2 and Th1 responses. This latter effect would explain why both allergic (Th2) diseases and auto-immune (Th1) diseases have both increased in relatively affluent communities and co-exist more frequently than by chance. However, while in some communities, active infection such as measles, and immunization with live organisms such as BCG, has been associated with less allergy, it is more likely that the commensal human microbiome has the most important influence in promoting normal immune regulation. This has resulted in the more accurate descriptor; “the microbial exposure hypothesis.” It is likely that changes in the commensal normal human microbiome in communities adopting a Western life-style have increased susceptibility to all non-communicable inflammatory diseases (115).

If we consider this microbial exposure hypothesis true, there is a problem with the interpretation of epidemiological cohort studies showing an increased frequency of respiratory infections in children who subsequently develop allergic sensitization and disease. The underlying defects in immune response increasing the risks of infection may be the same that increase the likelihood of allergy. This may thus imply that the infection/allergy association is not cause and effect.

However, there are certain predominantly infant viral infections that have been specifically associated with the later development of asthma. Rhino-virus (RV), the common cold virus (particularly with the RV-C) induced wheezing in infancy predicts a very high probability of later asthma (115). To a lesser extent this process has also been attributed to infant bronchiolitis due to RSV infections, although recent outcomes of the MAKI study in which infants in the first year of life were treated with palivizumab against RSV or not treated suggest that although palivizumab treatment resulted in a significant reduction in wheezing days during the first year of life, the incidence of asthma at 6 years of age did not differ between the groups (116, 117). In addition, it has long been known that acute gastro-enteritis in infancy increases the risk of allergic sensitization to food proteins, most notably from cow's milk, if the exposure to allergenic proteins occurs while there is still intense gut inflammation. The latter provides the co-stimulatory signals to trigger a sensitizing response (115). Thus breast-feeding with its known protective effects against GI and RSV infection will reduce such events. In addition the human milk oligosaccharides that are present in breast milk facilitate the development of a beneficial microbiome which through generation of short-chain fatty acids enhances T-cell regulation amongst a number of other beneficial effects (118).

### Bovine IgG and colostrum: effects on respiratory tract infections in preclinical models

Several *in vitro* and animal studies have shown effects of bovine IgG and colostrum in viral respiratory tract infections. Bovine immunoglobulins can bind to—and *in vitro* even neutralize—RSV, a common childhood pathogen resulting in upper respiratory tract infections in infants especially in the first year of life (38). Furthermore, bovine immunoglobulins from non-immunized cows have been shown to be able to bind to human RSV as well as to influenza virus, and are able to prevent the infection of Hep2 cells by human RSV *in vitro* (38). In addition, dietary bovine colostrum could reduce the severity of infection and viral titers in a murine model of RSV infection (119). Specificity of the antibodies against respiratory viruses might further enhance efficiency in prevention and/or treatment of disease as was discussed above for gastrointestinal pathogens. Indeed, bovine IgG isolated from hyperimmune colostrum, as well as F(ab')2 fragments thereof could prevent infection with influenza PR8 virus after intranasal application in mice (120). This indicates that Fc receptor-independent mechanisms such as neutralization or immune complex formation are the mechanism behind this finding. In a follow up study (non-immunized) oral bovine colostrum reduced the severity of influenza infection, by reducing viral load and preventing loss of body-weight (121). In addition, splenic NK activity, as well as the production of IgA producing B cells in the small intestine and lungs, was noted in the colostrum group. For RSV, however, this non-specific response might be less relevant since the F protein of bovine RSV has great similarities to human RSV (122) which explains the presence of specific antibodies against human RSV in bovine milk of non-immunized cows.

### Prevention of upper respiratory tract infections (urtis) and otitis media (OM) in children and infants with bovine IgG and colostrum

Epidemiological data previously showed that infants that receive unprocessed (raw) bovine milk as a weaning food in the first year of life had a lower chance to get respiratory infections and otitis media compared to infants that received ultra heat-treated (UHT) milk (28). A hypothesis is that the lack of intact proteins in UHT milk vs. the presence of these proteins in unpasteurized cow's milk explains the observed effect. Indeed, bovine IgG and colostrum have been reported to prevent upper respiratory tract infections in children, adults, elderly people and athletes (93, 94, 123–130) and these studies are summarized in Table 4.

**Table 4:** Prevention of URTI with bovine IgG and colostrum.

**Subject characterisitics**	**Type of study**	**Product, dosage, and duration**	**Reported outcome**	**References**
**INFANTS/CHILDREN**				
1–6 year old Egyptian children with recurrent URTI and/or diarrhea (*n* = 160)	open, non-comparative	Colostrum, 3 g/day for <2 year 6 g/day for >2 years, 4 weeks	Lower number of episodes and hospitalizations for URTI (and GITI)	(93)
1–8 y old Indian children with recurrent URTI/diarrhea (*n* = 605)	open, non-comparative	Colostrum 3 g/day, 12 weeks	Lower number of episodes and hospitalizations for URTI (and GITI)	(94)
5–17 year old Turkish IgA deficient infants with recurrent URTI (active *n* = 16; placebo *n* = 15)	Placebo controlled, randomized, double blind	Colostrum 3 × suckling tablet (incl 14 mg colostrum+2.2 mg lysozyme) /d, 3 days	Lower infection severity score in treatment group, no effect on salivary IgA	(123)
3–9 year old healthy japanese children (active *n* = 103; placebo *n* = 104)	Placebo controlled, randomized, double blind	Late colostrum (10% Igs)- vs. semi skimmed milk tablets 0.5 g/day. 9 weeks	Frequency and duration of URTI was lower in the treatment group vs. the control group, especially in the 3–6 year old children	(127)
3–7 year old Italian children with recurrent URTI (active *n* = 67; placebo *n* = 100)	Retrospective observational study	Sinerga (incl. colostrum, incl probiotics) vs. bacterial extracts 1 sachet (a 3 g)/day, 10 days month 1, 20 days months 2,3,4	Greater reduction in the frequency of respiratory infections that needed antibiotic therapy in the group of children supplemented with Sinerga than in the group treated with bacterial extracts.	(124)
**ADULTS**				
>18 years old australian adults with 3 or more URTI in the last 6 months (active *n* = 53; placebo *n* = 52)	Placebo controlled, randomized, double blind	Bovine lactoferrin /whey protein Ig-rich fraction (Lf/IgF) vs. placebo 2 × 300 mg/day, 90 days	# Cold events over 90 days significant lower in treatment group compared to control group. Duration and severity of cold was not different between groups.	(125)
14–27 year old Trained swimmers and age matched controls (4 groups) from New Zealand (active *n* = 25; placebo *n* = 28)	Placebo controlled, randomized, double blind	Low protein colostrum powder (3% Ig w/w) vs. isocaloric placebo 2 × 25 g/day, 10 weeks	A non-significant trend for lower upper respiratory symptoms in athletes consuming colostrum vs. placebo was reported. No effects in control group. No differences between groups wrt saliva and serum Igs.	(126)
18–50 year old adults in the UK (active *n* = 25; placebo *n* = 28)	Placebo controlled, randomized, double blind	Colostrum vs. iso-energetic/-macronutrient placebo 2 × 25 g/day, 10 weeks	Significantly lower proportion of days with URI during the 12 weeks in the COL group (5%) compared to the PLA group (9%). No difference on *in vitro* immune functionality, except for lower bacterial load in saliva of colostrum subjects.	(17)
25–30 year old trained Australian cyclists	Placebo controlled, randomized, double blind	Colostrum protein concentrate (CPC, 20% Ig) vs. whey protein 10 g/day, 8 weeks	Trend toward reduced incidence of upper respiratory illness symptoms in the bovine CPC group (*P* = 0.055). Diverse immune parameters changed in CPC group.	(131)
18–35 year old Australian healthy volunteers (active *n* = 93; placebo *n* = 81)	Placebo controlled, randomized, double blind	Colostrum protein concentrate (CPC) vs. whey protein 3 × 20 g/day, 8 weeks	Incidence but not duration of URTI was significantly lower in CPC group compared to whey group.	(128)
50–60 year old Italian healthy volunteers (*n* = 41 vs. *n* = 36 vs. *n* = 39 vs. *n* = 23)	Randomized study	Vaccination ± colostrum product vs. colostrum only vs. no prophylaxis 1 tablet a 400 mg (25-40% Ig)/day, 8 weeks	Number of days with flu was 3 times higher in the non-colostrum compare to the colostrum treated group (colostrum+vacc 14 vs. vacc only 57 vs. colostrum only 13 episodes vs. non-treated 41)	(129)
**ELDERLY**				
60–70 year old Italian elderly people with high risk for influenza (heart/lung problems) (*n* = 21 vs. *n* = 20 vs. *n* = 19)	Randomized study	vaccination ± colostrum product vs. colostrum only vs. no prophylaxis 1 tablet a 400 mg (25–40% Ig)/day, 8 weeks	The incidence of complications and hospital admission was higher in the group that received only a vaccination compared with the colostrum groups.	(129)

Five studies have shown a reduced incidence or severity of upper respiratory tract infections in children that received colostrum from non-immunized cows. Of these studies, two were double blind and the other 3 studies were prospective, open or uncontrolled. The first double blind placebo controlled study was performed in IgA-deficient children with viral upper respiratory tract infections that received a sucking tablet containing 14 mg of colostrum three times per day which lowered the infection severity score after 1 week (123). In another study, 195 children of 3–9 years old received either three colostrum or control milk powder chewing tablets per day for 2 months corresponding to 0.5 g colostrum or control milk powder per day (127). The children receiving colostrum tablets, especially in the 3–6 years age group, had a reduced upper respiratory tract infection frequency, compared to placebo, as well as a reduced number of reported sick days. A prospective open, non-controlled, study in children suffering from recurrent episodes of URTI or diarrhea were given powdered colostrum and showed a reduction of these infections compared to baseline (93). In another large, uncontrolled open study, significant decreases in diarrhea and upper respiratory tract infection (URTI) episodes were reported in Indian children receiving an oral colostrum for 12 weeks (94). The same was noted in a retrospective study comparing two groups of children, one that received a product containing colostrum plus a probiotic for 4 months, and the other that received a bacterial extract for 3 months (124). Children that received the colostrum/probiotic combination had a reduction in respiratory tract infections requiring antibiotic therapy compared to the children receiving bacterial extracts.

These studies suggest that bovine colostrum can prevent upper respiratory tract infections although the open, non-controlled prospective studies should be interpreted with care. In line with studies in children, the use of colostrum to prevent respiratory tract infections has also been studied in adults and elderly people. In adults suffering from frequent upper respiratory tract infections, supplementation with a bovine IgG and lactoferrin containing whey protein fraction (600 mg/day for 90 days) showed a reduced incidence of the common cold and cold-associated symptoms compared to the placebo group in a double blind randomized, placebo controlled study (125). Finally, decreased numbers of self-reported upper respiratory tract infections have also been noted after colostrum supplementation in athletes (126, 128, 130, 131), and colostrum prevented influenza infection in elderly volunteers comparable to influenza vaccination (129). In the latter study, colostrum was consumed for 2 months and flu episodes were scored for 3 months. Also, the effect of colostrum supplementation alone, colostrum combined with vaccination or vaccination alone was studied in a high-risk cardiovascular subject group on flu-associated complications in the hospital. Colostrum groups showed significant lower flu-associated complications compared to the vaccination only group.

Intervention studies aiming to reduce otitis media using bovine milk proteins such as immunoglobulins are scarce. Recently, an infant formula containing milk fat globule membrane components (proteins and phospholipids) reduced the incidence of otitis media in infants 2–6 months of age in a double blind placebo controlled study (132). Interestingly, a component of the proteins in this milk fraction are immunoglobulins (133).

Taken together, there is an increasing number of studies that suggest a role for bovine IgG or colostrum in preventing or ameliorating viral respiratory tract infections. To what extent this protective effect against infection will also affect allergy prevalence remains to be established. However, the reduced prevalence of allergic conditions amongst farming families in many countries (134) has been directly associated with the use of unpasteurized milk by pregnant and lactating mothers, and their infants after weaning (27, 135). Maternal consumption of farm dairy products during pregnancy resulted in reduced levels of pro-inflammatory cytokine production from stimulated cord blood mononuclear cells from the very large multinational European study (PASTURE) (136).

## Bovine immunoglobulins: putative mechanism of action

Bovine immunoglobulins can exert their effects on several levels: via direct effects on potential pathogens, via enhancing clearance of pathogens, via influencing intestinal barrier function, and via modulating immune function. This section will discuss the mechanistic evidence available to date.

### Direct effects of immunoglobulins toward pathogens

The primary role of immunoglobulins on mucosal surfaces is to bind to pathogens to prevent their entry into the body. This process is termed immune exclusion. During immune exclusion the pathogens as well as the immunoglobulins remain confined to the intestinal lumen and the immunoglobulins prevent adhesion to intestinal epithelium. Hyperimmune bovine immunoglobulins can prevent the adhesion of pathogens to intestinal epithelial cells (75) and even immunoglobulins from non-immunized cows can prevent adhesion of some pathogens (65, 137). As an example, the adhesion of *Clostridium difficile* to human intestinal epithelium cells (Caco-2 cells) was inhibited dose-dependently by normal bovine colostral whey (137) and spray dried colostrum from normal cows was shown to inhibit the adhesion of several necrotizing enterocolitis-associated pathogens to HT-29 colonic epithelial cells (65).

### Effects on barrier function and gastrointestinal inflammation

At the next level, there are indications that bovine immunoglobulins and colostrum can also support intestinal barrier function. When intestinal barrier function is compromised, bacterial products such as LPS, food allergens, as well as pathogens can passively cross the epithelial layer and cause inflammation and infection in the mucosa. Bovine colostrum can inhibit the NF-κB signaling pathway and induction of pro-inflammatory cytokines in HT29 cells, suggesting colostrum has direct anti-inflammatory effects on intestinal epithelium (138). Furthermore, bovine IgG can also have anti-inflammatory effects by preventing translocation of bacterial components across the epithelial layer. This was investigated in a co-culture model of intestinal epithelial C2BBe1 cells and THP-1 cells. In this model bovine serum derived IgG could prevent the translocation of bacterial components over the epithelium, thus preventing inflammatory responses to bacterial ligands in the underlying THP1 cells (139).

To further investigate the potentially mitigating role of bovine IgG in inflammatory responses, effects of immunoglobulins and colostrum have been addressed in human and animal models for necrotizing enterocolitis (NEC), irritable bowel syndrome (IBS), inflammatory bowel disease (IBD), non-steroid anti-inflammatory drug (NSAID)-induced gut damage and perioperative endotoxemia. IgG purified from hyperimmune colostrum could protect mice against TNBS-induced colitis (140). Mice receiving hyperimmune colostral IgG before colitis induction had reduced weight loss, improved histological score and increased serum IL-10 as well as increased numbers of Tregs compared to controls. Similar findings were reported for bovine serum IgG from non-immunized cows in a bacterial-induced colitis model (141), a chemotherapy-induced mucositis model (142) and a pathogen infection model (143). Likewise, serum-derived bovine IgG reduced mucosal expression of pro-inflammatory cytokines and prevented reduction of barrier function through preventing reduction of tight junction proteins ZO-1 in a mouse model for IBD (144). Bovine IgG has also been studied in IBS, where it improved symptom scores in IBS patients with recurrent diarrhea (145). In addition, oral bovine immunoglobulin induced some improvement in a subgroup of IBD patients (146).

Animal models indicate that colostrum may prevent NEC. Preterm piglets fed normal bovine colostrum had better growth and lower NEC incidence, NEC severity and intestinal cytokine production compared to piglets fed formula feeding (147, 148). The use of bovine colostrum is under investigation in preterm infants. In the safety phase of a pilot study bovine colostrum was well-tolerated in the pre-term infants, and further studies are ongoing (149).

Two studies have addressed the effect of immunoglobulin-enriched non-immune colostrum on peri-operative endotoxaemia as a measure for peri-operative infections. In the first study by Bölke et al. a reduced peri-operative endotoxaemia was noted after abdominal surgery, suggesting a stabilization of gut barrier function and neutralization of endotoxins by colostrum (150). A second study, however, could not confirm this effect after coronary bypass surgery, possibly because the dose used was lower (151). Finally, non-steroidal anti-inflammatory drugs (NSAID)-induced intestinal damage has been shown to be reduced after colostrum treatment in animal experiments (152) and NSAID-induced increases in intestinal permeability were reduced in human volunteers receiving colostrum (153). However, these effects are probably not mediated by bovine IgG but by TGF-β that is also present in colostrum (152).

These findings indicate that bovine IgG (probably in concert with additional colostrum components) prevents bacterial transfer and leakage of LPS over intestinal epithelium, modulates the expression of epithelial tight junction proteins and inhibits intestinal inflammation. This is comparable to findings in studies on the role of IgA in breastfeeding in animal models (154–157), suggesting that oral immunoglobulins from cows may have a similar mode of action as breastmilk-derived IgA.

### Immune effects

Immunoglobulins consist of an antigen binding domain (in the variable region of the molecule) and a constant region. This constant region is essential for inducing effector functions of immunoglobulins such as phagocytosis and clearance of pathogens, complement fixation, antigen presentation and immune regulation. These downstream effector functions of immunoglobulins are dependent on binding of the constant region to immunoglobulin Fc receptors (Fc receptors). These receptors are primarily expressed on the cell surface of immune cells. Secretory IgA (sIgA) does not have these effector functions because it is a dimer containing a secretory component, and therefore the constant part is not available for binding to receptors.

Interestingly, bovine IgG is able to bind to Fcγ receptors on human cells. bovine IgG was shown to bind to human monocytes, neutrophils and macrophages, as well as to B cells (158, 159). Coating (opsonisation) of *Streptococcus mutans* with bovine IgG induced phagocytosis and killing of the bacteria by human leucocytes (160). Similar findings were reported for *S. epidermidis* and RSV (38).

Bovine IgG inhibited pokeweed mitogen-induced secretion of immunoglobulins in PBMC cultures, probably mediated by FcγR as F(ab')2 fragments had no effect (161). This was later confirmed by another group, who suggested that bovine IgG binds to FcγRII, the only FcR expressed on B cells (159). Indeed, also binding of bovine IgG1 to FcγRII on phagocytes (monocytes and PMN) was shown by Jungi in 1989 and to be dependent on immune complexes (158). Now it is known that two types of FcγRII exist: The activating FcγRIIa, expressed on monocytes, dendritic cells and neutrophils, and the inhibitory FcγRIIb, mainly expressed on B cells and mast cells (162–164). Whereas, activating FcR contain an ITAM (immunoreceptor tyrosin activation motif), the inhibitory FcγRIIb contains an ITIM (immunoreceptor tyrosin based inhibitory motif) in its intracellular tail. When FcγRIIb is crosslinked, the downstream signaling of the ITIM leads to a inhibition of activating signals, resulting in dampening of the immune response, i.e. antibody production. On the other hand, binding of immune complexes or opsonized pathogens to activating FcR on phagocytes will lead to activation: phagocytosis, antigen presentation and cytokine production. The homology of the extracellular part of FcγRIIa and FcγRIIb is very high: 92%. However, human IgG2 binds to FcgRIIa but not to FcgRIIb (165).

The fact that bovine IgG can bind to human FcγRII was confirmed more recently (38, 166). These two research groups demonstrated directly that bovine IgG is able to form immune complexes with bacterial and viral pathogens, mediates FcγR-mediated uptake and antigen presentation, as well as phagocytosis and killing in phagocytes.

Less is known on the interaction of bovine IgG with other receptors. Bovine IgG1 can also bind to a non-classical receptor for IgG, the human neonatal Fc receptor FcRn, albeit with lower affinity compared to human IgG (167). FcRn is expressed on intestinal epithelium in humans throughout life (168, 169). However, evidence suggests that bovine IgG is not taken up into circulation of adult human volunteers (36, 63) and unpublished observations). It is therefore not clear at present if and what the consequence of binding of bovine IgG to FcRn is.

In contrast to short-lived passive immunity, adaptive immune responses occur during the lifetime of an individual as an adaptive response to specific infecting pathogens on subsequent exposures and in many cases, confer lifelong protective immunity to reinfection with the same pathogens. An adaptive immune response involves activation, selection, and clonal proliferation of T and B cells. After encountering an antigen, T cells proliferate and differentiate into antigen-specific effector cells, while B cells proliferate and differentiate into antibody-secreting cells.

Adaptive immune responses are initiated by activation of CD4+ T helper cells after antigen presentation by antigen presenting cells (APC). APC take up exogenous (protein) antigens from their environment via pinocytosis or receptor-mediated endocytosis, degrade them into peptides, and present these peptides in the context of MHC class II molecules to the T-cell receptor (CD3) on antigen-specific T cells (170).

Facilitated antigen presentation has been described for cell surface expressed Fc receptors on professional antigen presenting cells. Fc receptors can internalize bacteria and viruses as well as protein antigens that are bound by circulating serum immunoglobulins, resulting in killing of pathogenic microorganisms, and antigen presentation to initiate immune responses.

Low- and intermediate affinity Fc receptors have a low affinity for monomeric immunoglobulins, but have a high affinity for immune complexes. As a result, these receptors will only bind immunoglobulins when they are in complexes together with the antigens they recognize. Indeed, for Fcγ receptors (171, 172), Fcα receptors (173) and Fcε receptors (174, 175) it has been shown that immune complexes are taken up more efficiently by antigen presenting cells than the antigens alone. These studies demonstrate that antigen-specific responses of CD4+ T helper cells are facilitated strongly by specific antigen uptake via Fc receptors on antigen-presenting cells, resulting in more efficient immune responses. This is especially relevant when low antigen doses are present.

In line with this a recent study shows increased CD8+ T cell activation in mice infected with RSV when they received bovine colostrum orally (119). Therefore, in addition to immune exclusion by direct binding of bovine IgG to pathogens, FcγRII mediated pathogen clearance and killing by macrophages and neutrophils, bovine IgG may also play a role in enhancing adaptive immune responses by increasing T and B cell responses to pathogens.

**Figure 2:**
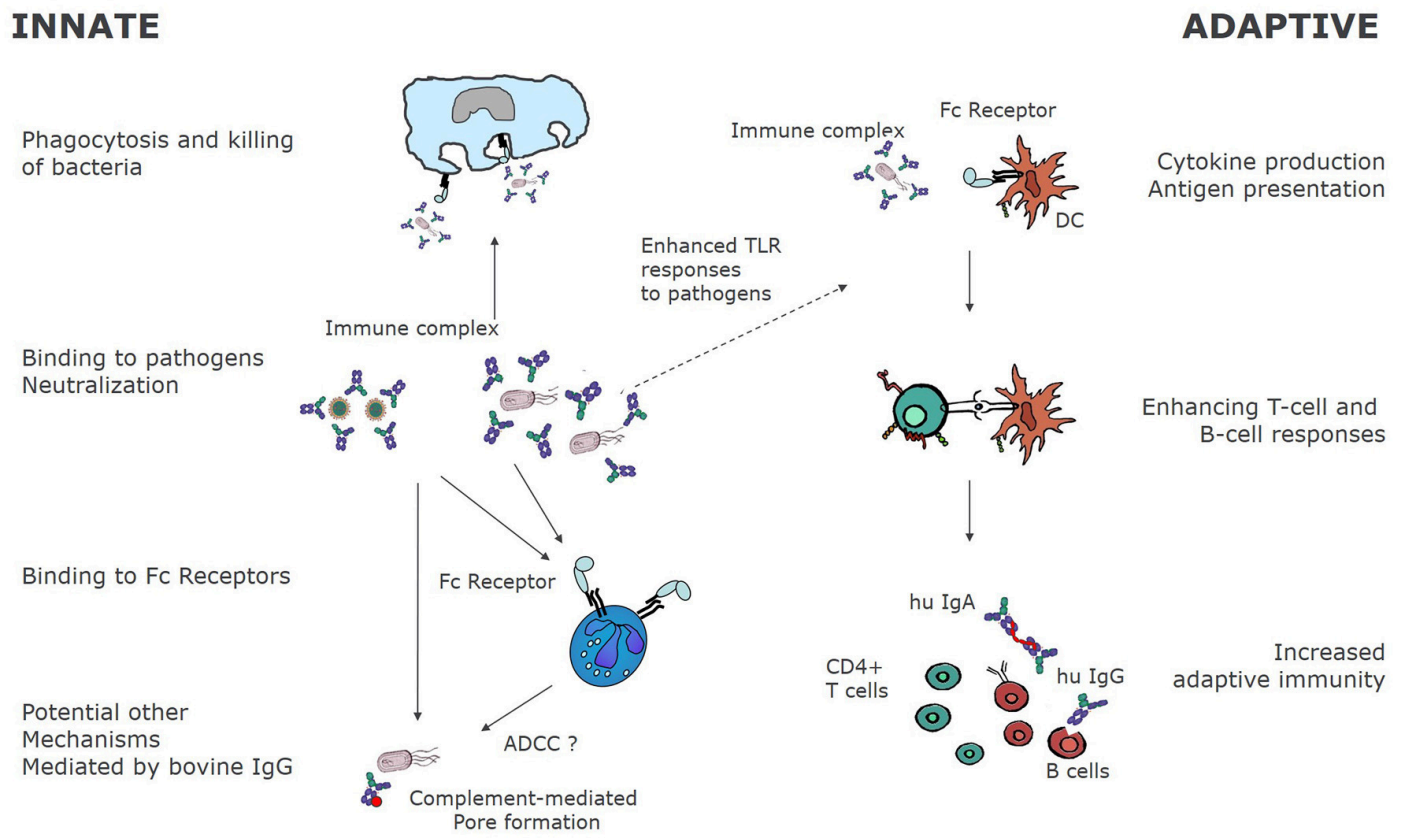
Immunological mechanisms of bovine immunoglobulins. Bovine immunoglobulins can modify innate as well as adaptive immunity. By binding directly to pathogens, bovine immunoglobulins can bind to FcγR bearing innate immune cells, leading to phagocytosis and killing. In some cases, as for RSV, bovine IgG may also fully neutralize human pathogens as demonstrated for RSV *in vitro* (38). Other mechanisms of pathogen elimination may be complement mediated pore formation and killing, and ADCC. On the other hand, as bovine IgG-pathogen immune complexes bind to FcγR, receptor mediated uptake and antigen processing is enhanced, resulting in increased T, and ultimately B-cell responses to the pathogens.

Experiments in murine animal models have also found increased NKT in mice that ingested colostrum containing LPS-specific bovine IgGs (176). Similarly, increased NK activity was reported in spleen and Peyer's patch of mice that ingested bovine hyperimmune colostrum (121, 143, 177). It is not clear at present if these NK cells are induced by IgG or by other factors present in colostrum.In summary, Figure 2 shows the mechanism of action of bovine IgG on the immune system.

### Immunological effects of oral immunoglobulins of other species

In addition to oral bovine immunoglobulins, several studies with murine, porcine and human oral immunoglobulins have elucidated mechanisms that may also occur after ingestion of bovine immunoglobulins.

A series of recent studies in mice have shown yet another potential mechanism for the presence of immunoglobulins in the intestinal lumen. These studies have shown that locally produced intestinal IgA can bind to intestinal bacteria and modify the microbiota composition as well as immune responses toward bacteria in the gut (178–184). These studies suggest that oral immunoglobulins may also have an effect on microbiota development and composition.

Indeed, maternal sIgA in breast milk was shown to significantly modify microbiota composition at weaning and later in life, limit translocation of pathogens in suckling pups and ameliorated DSS induced colitis (154). In addition, IgG2 and IgG3 as well as sIgA in breast milk were shown to bind to commensal bacteria, dampen mucosal T cell activation, and prevent bacterial translocation to the mesenteric lymph nodes (155). Similar findings were reported previously, showing that both sIgA and IgG in breast milk can play a role in immune exclusion as well as in dampening T cell responses to intestinal bacteria (156, 157).

Similar effects of IgG in breast milk of mice have been obtained in a study in which immune complexes of murine breast milk IgG and allergens prevented the development of experimentally induced asthma (185). IgG-allergen immune complexes were transferred from breast milk into the suckling pups via FcRn, and induced FoxP3+ regulatory T cells that were needed for the protective effect on asthma development. This was recently confirmed in another study (186). Interestingly, in humans this process also occurs, but in humans it occurs when IgG-allergen complexes cross the placenta. This results in much lower rates of infant sensitization to that allergen (187).

As described above, bovine milk contains IgG directed against inhalation allergens (41). The functional relevance and possible applications of such allergen-specific bovine immunoglobulins are not clear at this time, but they may play a role in the protective association of raw milk consumption with reduced prevalence of allergies (4).

Immune complexes of maternal breast milk immunoglobulins with maternal GI microbiota components have been shown to be crucial for delivering tryptophan-derived AhR ligands that promote barrier function in the suckling pups in an IL-22 and Innate Lymphocytic Cell type 3 (ILC3)-dependent manner (188).

Oral ingestion of porcine immunoglobulin also contributes to prevention of inflammatory tissue damage in LPS-induced acute lung inflammation in mice. Oral IgG enhanced the expression of IL-10 producing Treg, decreasing Th1 and Th2 cytokines and decreasing chemokine production in the lung in response to LPS (189).

These studies indicate that oral immunoglobulins may help to regulate immune responses to the microbiota as well as allergens—on the one hand promoting immune exclusion of pathogens, and on the other hand preventing excessive immune responses to commensal bacteria. Studies on effects of bovine Ig and colostrum ingestion on fecal microbiota composition in infants during colonization of the GI tract have not been performed to date, but based on these findings it may be expected that oral intake of bovine IgG may similarly affect microbiota composition, allergy and immune development in humans.

## Concluding remarks

The protective role of IgA in breast milk is well-documented. IgA protects infants against infection, shapes the microbiota and creates a non-inflammatory response against the microbiota, thus preventing intestinal inflammation.

As described above, bovine IgG has a number of similar effects: binding to human-relevant pathogens, effects on phagocytosis mediated through Fc receptors for human IgG and prevention of infection in human studies. So functionally, bovine IgG does have effects on the human immune system beyond just binding to potential pathogens. In addition, especially in young infants, IgG does seem to pass through the gastrointestinal tract without being fully degraded by digestion, thus leaving its functional aspects intact. As the fine specificity as well as the effector functions of bovine IgG are not identical to breast milk IgA (and IgG), one question that remains to be answered in clinical studies is if inclusion of functionally active bovine immunoglobulins in infant nutrition can fully restore the lack of maternal IgA in bottle fed children.

Immune support by bovine immunoglobulins as an alternative for breast milk-derived IgA is especially relevant in the period just after birth in case the mother cannot breastfeed her child and is dependent on bottle feeding, but also in the weaning period at around 4–6 months when respiratory and gastrointestinal infections are known to increase as the passively acquired maternal IgG in serum, as well as the maternal breast milk-derived IgA in the gastrointestinal tract of the infant, have decreased to very low levels.

It should be stated though, that the increased prevalence of infections after weaning is not completely related to deprivation of IgA but is also linked to the introduction of new foods, an increased exposure to the outside world and in part by the absence of additional protective factors in breast milk.

Currently, formula fed children receive no or very low levels of functionally active bovine immunoglobulins. This is because current foods processing technologies as well as legislation strongly depend on heating as the technique of choice to achieve microbiological safety. Overcoming these challenges by applying novel food processing technologies may enable the application of bovine immunoglobulins in infant nutrition.

Future research will be needed to confirm if this will indeed result in a reduction in gastrointestinal and respiratory infections, and if this will also be associated with a decreased prevalence of asthma that is linked to early respiratory tract infections in infants.

## Author contributions

LU, JL, HS, JW, and RvN all contributed to the writing of the manuscript.

### Conflict of interest statement

LU and RvN are employees of FrieslandCampina. JW has been the PI for a trial of a cow milk formula with prebiotics for the prevention of allergy funded by Danone/Nutricia. JL, HS have no conflicts of interest to report. The remaining authors declare that the research was conducted in the absence of any commercial or financial relationships that could be construed as a potential conflict of interest.
